# Assessment of Undernutrition in Pediatric Chronic Kidney Disease - Gaps and Opportunities

**DOI:** 10.3389/fped.2022.866498

**Published:** 2022-05-12

**Authors:** Arpana Iyengar, Robert H. Mak

**Affiliations:** ^1^St John's Medical College Hospital, Bangalore, India; ^2^Rady Children's Hospital, University of California, San Diego, San Diego, CA, United States

**Keywords:** nutritional assessment tools, diet, appetite, PEW, SGNA, body cell mass, whole body potassium counter, muscle strength

## Introduction

Optimal nutrition is critical to meeting twelve of the sustainable developmental goals relevant to non-communicable diseases such as kidney health and survival for 2030 (1). These goals, as proposed by the United Nations, are the universal call to action to end poverty, protect the planet, and improve lives globally. Chronic Kidney disease (CKD)-related nutritional disorders consist of 20–45% of undernutrition and 15–30% of overnutrition (2, 3). The challenge lies in the fact that no single measure can comprehensively reflect the underlying nutrition status. The Pediatric Renal Nutrition Taskforce clinical practice recommendations allude to the need to further explore the scope of various assessment tools (4). Here, we put forth the gaps and opportunities of selective nutritional assessment tools in children with CKD, as an impetus to stimulate further research questions.

### Dietary Assessment Methods

The traditional approach to dietary assessment includes a diet history, diet diary, and a food frequency questionnaire (FFQ) (4). The clinician should be mindful of the merits and limitations of these methods. A 24-h multiple diet recall for at least a 3-day period (including weekdays and weekends) is the preferred method for children aged 4–10 years and has been used commonly in studies on children with CKD. This captures the frequency of intake and cooking methods, while underreporting and inaccurate portion size reporting are limitations. A diet diary records food intake over a week and is considered a gold standard approach (especially used in younger children) as there is no recall burden and captures variation in food by weight or portion size. The FFQ approach captures the frequency of food and drink consumption over a given time period from a list of foods. This method is useful for diet assessment for special food and population groups, portion size, and micronutrients.

Technology-based dietary assessment is a promising, low cost, and real-time tool for the use at both individual and population levels (5). These online assessments use the same approach as standard diet history, FFQ, or diet records. A recent systematic review reflects good validity of web-based and mobile-based tools for dietary assessment compared to paper-based assessments especially in adolescents (6). Though not specifically studied in children with CKD, there is growing evidence on the utility of image-based and web-based assessment methods among children in general (7–9). However, smart phone applications and technology-based tools come with their own limitations of acceptability, responsiveness, accuracy, and consistency. The COVID-19 pandemic has rekindled such opportunities. In the context of nutrition-related research, best practice guidelines on the appropriate choice of diet assessment tools are available (10).

### Appetite, Taste, and Smell Assessment

Reduced appetite is observed in children with CKD and has shown to be associated with protein-energy wasting (PEW) and quality of life (11, 12). Clinical assessment of appetite, taste, and smell can help to identify specific eating behaviors that can potentially tailor dietary interventions. Most studies on appetite in children with CKD have used a 5-point rating scale (very good, good, fair, poor, and very poor) (11, 13). This approach was extrapolated from a study undertaken in adult patients on hemodialysis, wherein appetite rating was associated with decreased dietary energy and protein intake as well as mortality (14, 15). Psychometric appetite assessment tools specific to children have been used in high-income countries. A commonly used validated tool is the Children's Eating Behavior Questionnaire (CEBQ) that has 35 items evaluated on a 5-point scale. Domains assessed include food or satiety responsiveness, slowness in eating, food fussiness or enjoyment, emotional undereating or overeating, and desire for drinks (16). Another tool developed for children under the age of 5 years relevant to low-income countries is the Early Childhood Appetite and Satiety Tool (ECAST) (17). Other recently studied tools include the picture-based appetite assessment for younger children aged 4–10 years (using pictures of individualized activities over desire to eat) and a visual analog scale (the patient marks the point of current state perceived on a line with two extreme states of appetite) for children above the age of 8 years (18). Abnormality in taste perception has been described in children with CKD that results from a low density of papillae and taste loss that strongly correlates with renal function (19). Taste and smell assessment is based on the pictural interpretation in children above the age of 5 years (20). The impact of COVID-19 on taste and smell adds further challenges. However, these tools have been studied for the research purpose and are not well utilized in routine clinical practice.

### Protein-Energy Wasting (PEW)

This is a specific entity used to identify severe forms of undernutrition in CKD. The diagnostic criteria for children, that is adapted from adult criteria, include five parameters: body mass index, mid-arm circumference (MAC), biochemical measures (serum albumin, cholesterol, transferrin, C-reactive protein), reduced appetite, and short stature. To diagnose PEW in children with CKD, anthropometry measures have been observed to be more useful than biochemical parameters (12, 21). Serum cholesterol and serum transferrin do not help in defining PEW in children with CKD. Serum albumin though highly prevalent is not associated with PEW in children with CKD. However, there are concerns regarding the cutoff value for serum albumin (at 3.8 g/dl) that is adapted from the adult criteria. There seems to be an opportunity to explore the role of a lower threshold of serum albumin (<2.1 g/dl) that is associated with high mortality rates in children with CKD in the diagnosis of PEW (20, 22). The existing studies on the utility of PEW reveal the need to reframe the present criteria.

Mid-arm circumference is a useful tool to diagnose undernutrition in general pediatric population. MAC is the circumference of the arm at the midpoint between the olecranon and acromion. In a large study of about 10,000 children (including those with underlying kidney disease), the sensitivity of MAC to identify children with no malnutrition was reported to be high at 92%. The specificity of MAC to detect severe malnutrition was 99% but the corresponding sensitivity reduced to 30% (23). MAC was noted to be strongly associated with the presence and severity of PEW in children with CKD and those on dialysis (12). MAC has also been used to characterize frailty in children with CKD along with other parameters of body mass index, C-reactive protein, and fatigue (24). MAC reflects subcutaneous fat tissue, bone, and muscle while mid-arm muscle area (MAMA) derived from MAC gives an estimation of the area of muscle portion of the arm excluding the bone. The role of MAMA (derived from MAC as MAMA(mm^2^) = [MUAC (mm) − Π [T]^2^/ 4Π where in T stands for triceps skinfold thickness, could be explored as a risk predictor of PEW. In adults with CKD, low-cost direct clinical assessments of muscle wasting are being explored. A study on ultrasonography measurement of muscle thickness of the quadriceps in adults on hemodialysis has shown promising results in detecting risk for PEW (25).

### Subjective Global Nutritional Assessment Tool

This assessment includes both nutrition-focused medical history and physical examination to identify malnutrition (undernutrition). Although s*ubjective global nutritional assessment tool* (SGNA) is recommended as a valid tool for nutrition assessment in adults with CKD and dialysis, similar studies in children are very limited (26). SGNA consists of 10 parameters (7 items in medical history and 3 in physical examination) to screen for undernutrition (27). Medical history lists out domains of anthropometry, dietary intake, gastrointestinal symptom, functional capacity, and metabolic stress of disease. Physical examination consists of assessment for muscle wasting, subcutaneous fat loss, and the presence of edema. A recent study undertaken on SGNA in children on dialysis revealed that out of the ten parameters, only five (anthropometry, diet intake, functional capacity, subcutaneous fat loss, and muscle wasting) were strongly associated with the presence and severity of malnutrition (28). Besides, SGNA had a poor agreement with objective measures of nutrition (MAC and serum albumin). SGNA could not pick up a change in nutritional status on the 8-month median follow-up in these children. This provides a rationale to reframe the SGNA rating form in addition to studying the scope of SGNA as a long-term monitoring tool. The role of nutrition-focused physical examination that demonstrates signs of muscle wasting and subcutaneous fat loss needs to be studied as the risk predictors of PEW and outcomes in children.

### Dual X-Ray Absorptiometry (DXA)

**Figure 1:**
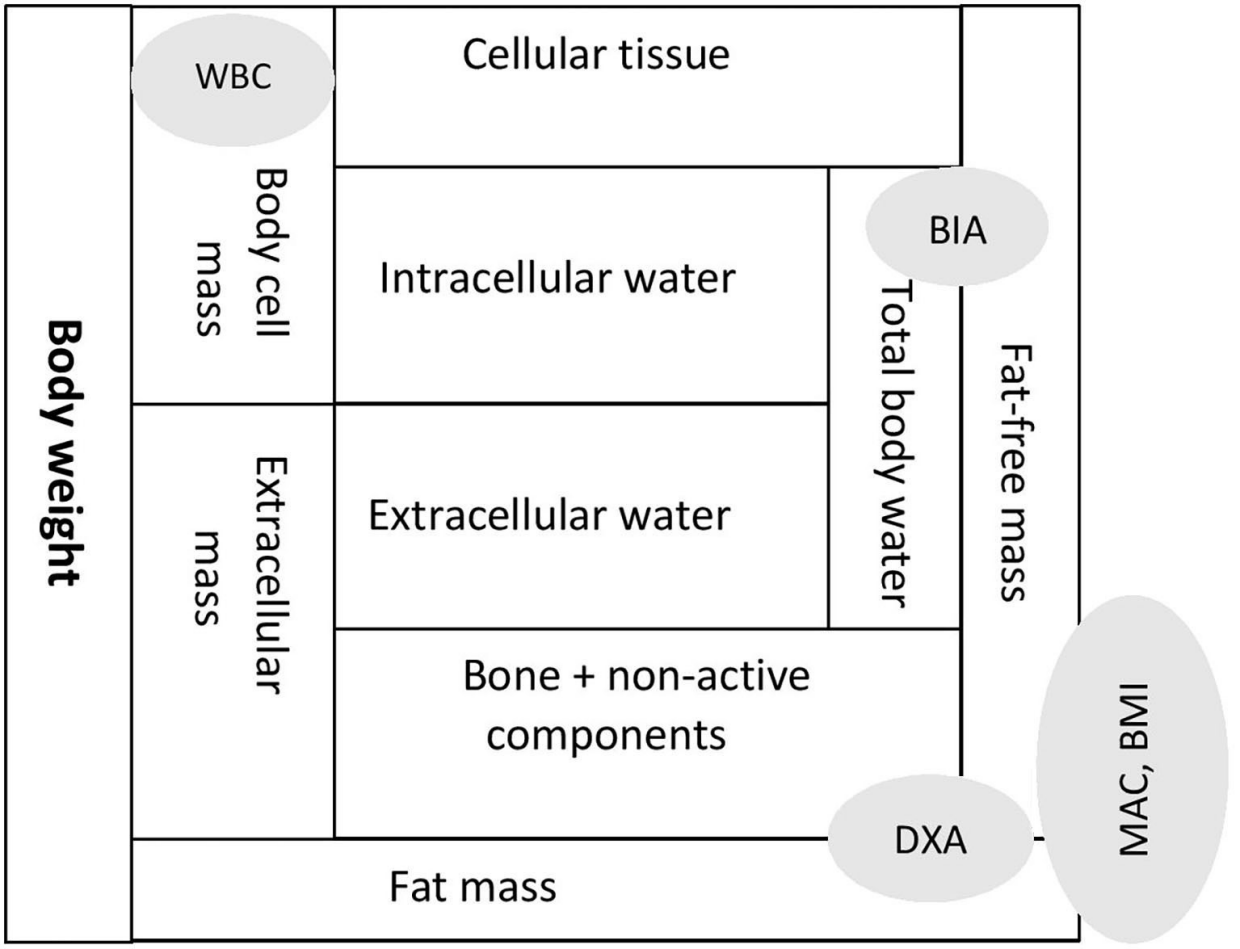
Body composition expressed as compartments and assessment tools reflecting specific compartments. MAC, mid arm circumference; BMI, body mass index; DXA, dual X-ray absorptiometry; BIA, bio-impedence analysis; WBC, whole body potassium counter.

Body composition is best understood by identifying compartments that constitute body weight (Figure 1). Anthropometry parameters (MAC and BMI) are the surrogate measures of fat mass (FM) and fat-free mass (FFM) compartments. *Dual X-ray absorptiometry* (DXA) and bioimpedance (BIA) methodologies measure FM and FFM compartments. However, FFM compartment, often equated as lean mass (LM), is affected by the hydration status, which is an important variable in CKD. A refinement for DXA in CKD is the measurement of appendicular LM, which provides a better solution to the hydration confounder. Another important limitation of some prior studies of body composition in children with CKD was a failure to account for the substantial differences in height and pubertal status between healthy children and those with CKD. A study on whole body and regional lean mass (LM) and FM across the spectrum of CKD severity found significant deficits in leg LM, indicating skeletal muscle wasting in children with moderate to severe CKD (29).

### Bioimpedance Vector Analysis

Bioimpedance analysis (BIA) is useful to detect total body water (both extra-cellular and intra-cellular water) and FFM. Body cell mass (BCM) is composed of cellular tissue and intra-cellular components and is the body's actively metabolizing, oxygen-consuming compartment. Tools that can measure BCM are bioimpedance vector analysis (BIVA) derived from BIA and total body potassium estimation through a whole-body potassium counter (WBC).

*Bioimpedance analysis* (BIA) when compared to the reference tool dual X-ray absorptiometry (DXA) demonstrated a poor agreement for the assessment of body fat and fat mass in children with CKD (30). Besides, predicting LM by absolute BIA measures is known to be inaccurate due to the influence of altered hydration in children with CKD. An alternative qualitative approach, derived from BIA, is the BIVA originally described by Piccoli (31). Recent studies discuss the utility of BIVA to assess body composition in children (32). Here, the resistance (R) and reactance (Xc), each adjusted for height (H), are plotted on “R/H-Xc/H” graphs. Interpretation is carried out by creating reference ellipses with regional data from the healthy population. BIVA is a dynamic tool as it indicates the level of hydration alongside the status of BCM. Studies on the utility of BIVA in adult patients with kidney failure are available whereas similar studies in children are scarce (33, 34). BIVA has shown to be useful in targeting dry weight in children on hemodialysis (35). Similarly, in children on chronic peritoneal dialysis, shifts across quadrants of cell mass and hydration can be tracked longitudinally by BIVA (36).

### Muscle Strength

Measuring handgrip strength is a simple, objective, and non-invasive bedside tool used to assess muscle function. Measurement is undertaken with a hydraulic dynamometer, and interpretation of values is based on z-scores derived from normative reference charts. It has proven to be an inexpensive, prognostic biomarker of stratifying cardiovascular mortality in a large prospective adult population study (37). In children, there is emerging evidence of impaired muscle strength in those with CKD with an underlying non-glomerular disease in particular, and impaired muscle strength noted is independent of growth retardation or body mass index (38, 39). However, unlike in adults, a strong association of physical activity to muscle strength in children with CKD has not been well established.

### Whole-Body Potassium Counter

Body cell mass, the functional mass in the body, contains 98% of potassium within the cells. The WBC is based on the novel principle of measuring the naturally occurring isotope of potassium (K^40^) present within cells, thus reflecting the BCM (40). Research on measuring BCM is not new as initial studies date back to 5 decades ago. Very few studies have explored the utility of measuring total body potassium for BCM in children with CKD with inconsistent findings (41–43). With the advances in technology, the decade just gone by has witnessed a new system of WBC that is safe, independent of tissue hydration and has reopened opportunities for research (44).

## Discussion

The International Society of Nephrology-Global Kidney Health Atlas, through a multinational survey, recently revealed the challenges pertaining to undertaking formal nutritional assessment in patients with CKD around the globe (45). This reinforces the need for nephrologists to have a good understanding of the utility and contextual interpretation of various nutrition assessment tools (Table 1). Translational research for more accurate and easily accessible biomarkers with confirmatory population studies represents an urgent medical need, especially in populations and regions where undernutrition is highly prevalent, and nutritional assessment by qualified personnel is a challenge.

**Table 1:** Merits and limitations of nutrition assessment tools for undernutrition in children with CKD.

**Assessment tool**	**Merits**	**Limitations**
**Diet** 3 day recall	- Easy to implement at patient level - No literacy skills needed - Provides details on frequency, portion size, and cooking methods/habits - Multiple 24h recalls possible	- Underreporting - Needs a qualified interviewer - Time consuming - Inaccuracies in recall
Diet diary	- Reference method - No recall needed - Can be reproduced - Minimizes inaccuracies	- Burden on parents - Needs literacy skills - Prone to change in food pattern or incomplete recording of food and drink - Needs data analysis software
Food frequency questionnaire	- Ideal for use at community level - Reflects food consumption patterns - Recommended for assessment of specific foods/ nutrients - Easy to collect information and details on portion size	- Demands accurate reporting - Time consuming - Needs literacy skills
Technology based	- Low cost, real time - Useful at individual and population level - Attractive to young patients - Less misreporting	- Acceptability, accessibility, accuracy, and response rate are the concerns - Not widely studied
Appetite Taste Smell	- Easy to implement and assess - Methods available based on age groups - Objective and image based methods are available	- Risk of subjectivity - Difficulty in comprehending rating scale terms - Not well studied - Appetite, taste, and smell are interdependent
PEW (Protein-energy wasting)	- Comprehensive assessment with 5 parameters - Provides status of both nutrition and inflammation - Focuses on appetite - Could be useful to track nutrition status over time - Highlights the utility of mid-arm circumference	- Needs blood sampling - Not widely studied in children - Existing evidence finds biochemical parameters not useful - Few parameters are extrapolated from adult definitions
SGNA (Subjective global nutrition assessment)	- Comprehensive tool incorporating nutrition-focused history and physical examination - Considers functional capacity, metabolic stress - Can be undertaken by a nurse/ health worker	- Not well studied in children - Diagnostic utility of certain parameters needs to be addressed - Longitudinal monitoring of nutrition status not a strength
DXA (Dual energy X-ray absorptiometry)	- Ideal to assess fat mass - Appendicular lean mass can be estimated	- Expensive, not a bedside tool - Needs normative age/gender/height specific data - Exposure to radiation - Overestimates lean mass in overhydrated state
BIVA (Bioimpedance vector analysis)	- Bedside tool at both individual and community level - Provide status of both hydration and nutrition - Potential to track dry weight, nutrition status long term	- Needs normative gender-specific ellipse graphs - Not well studied in children
WBC (Whole-body potassium counter)	- No radiation - Objective assessment of body cell mass - Noninvasive - Advanced technology based counters available	- Not a bedside tool - Time-consuming in young children - Limited availability of the tool - Overestimation of body cell mass in states of hyperkalemia

## Author Contributions

AI contributed to the manuscript writing. RM added intellectual inputs and edited the manuscript. Both authors contributed to the article and approved the submitted version.

## Conflict of Interest

The authors declare that the research was conducted in the absence of any commercial or financial relationships that could be construed as a potential conflict of interest.

## Publisher's Note

All claims expressed in this article are solely those of the authors and do not necessarily represent those of their affiliated organizations, or those of the publisher, the editors and the reviewers. Any product that may be evaluated in this article, or claim that may be made by its manufacturer, is not guaranteed or endorsed by the publisher.
